# A Spectral Filter Array Camera for Clinical Monitoring and Diagnosis: Proof of Concept for Skin Oxygenation Imaging

**DOI:** 10.3390/jimaging5080066

**Published:** 2019-07-26

**Authors:** Jacob Renzo Bauer, Arnoud A. Bruins, Jon Yngve Hardeberg, Rudolf M. Verdaasdonk

**Affiliations:** 1 The Norwegian Colour and Visual Computing Laboratory, Norwegian University of Science and Technology (NTNU), 2815 Gjøvik, Norway; 2 Department of Anesthesiology, VU University Medical Center, 1081 HV Amsterdam, The Netherlands; 3 Biomedical Photonics and Imaging Group, Faculty of Science and Technology, University of Twente, 7522 NB Enschede, The Netherlands

**Keywords:** spectral filter array, multi-spectral imaging, skin, bio-medical optics, occlusion measurement, reflectance spectroscopy, oxygenation, Xispec

## Abstract

The emerging technology of spectral filter array (SFA) cameras has great potential for clinical applications, due to its unique capability for real time spectral imaging, at a reasonable cost. This makes such cameras particularly suitable for quantification of dynamic processes such as skin oxygenation. Skin oxygenation measurements are useful for burn wound healing assessment and as an indicator of patient complications in the operating room. Due to their unique design, in which all pixels of the image sensor are equipped with different optical filters, SFA cameras require specific image processing steps to obtain meaningful high quality spectral image data. These steps include spatial rearrangement, SFA interpolations and spectral correction. In this paper the feasibility of a commercially available SFA camera for clinical applications is tested. A suitable general image processing pipeline is proposed. As a ’proof of concept’ a complete system for spatial dynamic skin oxygenation measurements is developed and evaluated. In a study including 58 volunteers, oxygenation changes during upper arm occlusion were measured with the proposed SFA system and compared with a validated clinical device for localized oxygenation measurements. The comparison of the clinical standard measurements and SFA results show a good correlation for the relative oxygenation changes. This proposed processing pipeline for SFA cameras shows to be effective for relative oxygenation change imaging. It can be implemented in real time and developed further for absolute spatial oxygenation measurements.

## 1. Introduction

Visual inspection of skin can provide physicians with diagnostic information about the patient. Inflammations, nutrition delivery, oxygenation, blood perfusion and other health indicators can affect the skin tone. In the Operating Room (OR) this information and especially the oxygen delivery is used by anesthesiologists as an early indicator. After light is reflected from the skin, it contains information of physiologic processes within the skin. Cameras can be more sensitive and capable to transfer this information into qualitative or even quantitative data. Analysing the relative changes in color or spectral reflectance over time allows monitoring of physiological processes.

Wieringa et al. [1] demonstrated the feasibility of using RGB cameras for oxygenation measurements analysing the ratio between the individual R, G and B color bands. Van Gastel et al. [2] extended RGB sensors with high temporal resolution for a camera based pulse oximetry system including a motion artifact resistant method. However, RGB camera sensors have a low spectral resolution with only three wide spectral bands, therefore the applications of monitoring vital functions is limited.

Multispectral imaging, on the other hand, is a technique to measure different narrow spectral bands. This allows a more accurate acquisition of the color or spectral changes in the reflectance of objects spatially. Spectral imaging has been applied in different areas and for different pathologies in the medical field. Some examples are the determination of bruise age [3,4], pigment mapping [5,6], melanoma screening [7] and burn wound analysis [8,9]. These imaging techniques are usually based on a temporal decomposition of the spectral bands with, that is, liquid crystal tuneable filters, filter wheels [10] or monochrome cameras with changing illumination. These techniques acquire different spectral bands at different time instances, therefore they are less suitable for quantifications of dynamic processes like oxygenation of skin. Nevertheless these techniques have been successfully applied to provide a spatially resolved measure of oxygenation [11,12,13].

Due to recent technological progress in interference filter design, a new multispectral imaging modality acquiring all spectral bands at the same instance has been developed. These spectral filter array (SFA) cameras combine the speed of commonly used RGB imaging systems with spatial and spectral images of the scene. A filter mosaic with multiple specifically selected spectral [14] transmission bands on top of an imaging sensor is the basis for this technology. The filters can for instance be based on Fabry-Pérot interference filters [15], which pass only specific wavelength bands at a given subpixel [16]. The bands can be chosen to provide a good spectral sampling of the scene in real time [17].

Additionally to the adaptation of these sensors in academia [18,19,20,21,22,23,24] some commercially available products have been developed by PIXELTEQ [25], SILIOS Technologies [26] and IMEC [27]. Acquisition speed, ease of use and versatile spectral range makes SFA cameras interesting for the medical sector, especially for the quantification of dynamic processes [28]. Filter wheel cameras or LCTF cameras on the one hand sample the spectral bands temporally SFA cameras, on the other hand sample the spectral information spatially. These spatially arranged filter sensitivities often come with a cost regarding the spectral sensitivities. Unlike the temporal spectral imaging systems, which have very narrowband filter sensitivities, some commercially available SFA imagers have double lobes in the spectral sensitivities. This unique architecture makes specific processing steps, like spatial decomposition, spectral calibration and the careful choice of optimal exposure necessary [15,20,24,29,30]. These can cause practical hurdles for clinical implementations of SFA imaging systems.

Oxygenation of tissue is a dynamic process and spatial oxygenation measurements in real time are of clinical value [31]. Temporal and spatial color changes can be used to provide indication of the micro-circulation of skin and oxygen distribution to tissue. The oxygenation of skin varies over its surface area as shown in previous studies [32] and therefore, spatial analysis is advantageous.

In this study the processing steps needed to make an SFA camera setup suitable for medical applications are developed and tested. A transferable basic processing pipeline for SFA cameras in the context of skin imaging is proposed. The focus is to maintain the full spectral range, ease of use of the imaging setup and the acquisition speed provided by this new technology. The proposed processing maintains the temporal, spectral and spatial attributes of SFA imaging systems, while solving technical hurdles introduced with this technology. A proof of concept on dynamic oxygenation distributions in volunteers using the spectra obtained with the SFA camera is conducted. The results are compared to a clinically validated device (gold standard) for oxygenation measurements and show relatively good sensitivity for oxygenation measurements.

## 2. Theoretical Background

**Figure 1:**
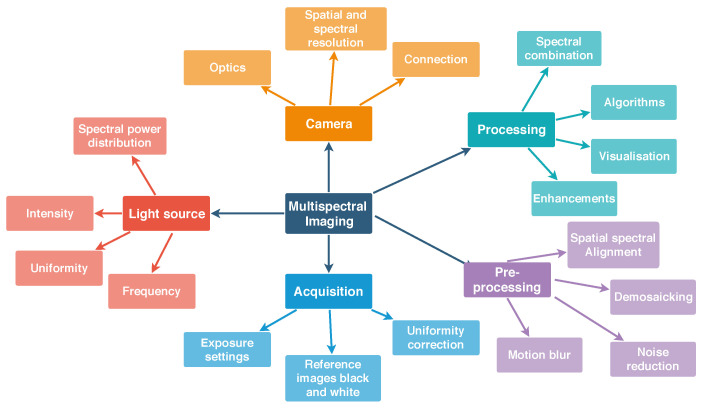
Overview of key aspects for generic spectral image acquisition including the camera, light source, acquisition, pre-processing steps and processing steps for the spectral image. For SFA imaging temporal aspects are of special importance: the flicker frequency of the light source and the connection speed to the camera. All of the items shown in this diagram have to be addressed for successful spectral image acquisition and meaningful data processing.

Key aspects to consider for the acquisition of multispectral images are presented in Figure 1. Most important aspects include the unique power distribution of the light source, the camera parameters for acquisition, pre-processing steps and finally the processing of the sensor data or the spectral image cube. The spectral image cube is the final output of most spectral imagers and each slice of the cube is an image sensed with a particular wavelength. A pixel in the plane of the spectral image cube represents the spectrum in that particular location. Table 1 shows advantages and disadvantages of different spectral imaging approaches [20].

SFA implementations provide a good tradeoff between true simultaneous snapshot capture of all wavelength, higher number of spectral bands, medium costs, ease of use and acquisition speed in terms of frames per seconds (fps).

Several processing steps are needed to obtain the spectral cube from an SFA image, while maintaining all of the benefits mentioned.

### 2.1. Light Skin Interactions

**Figure 2:**
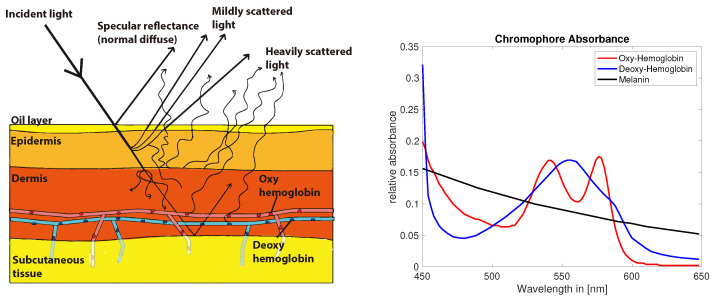
Skin light interaction simplified model (**left**) [43]. Absorption of common chromophores in the visual range of the light spectrum (**right**) (data compiled from [44], Figure recreated from [43].).

Skin color contains physiologically relevant information and optical or visual inspection of skin is a commonly used method in the medical practice. The perceived color of skin is the resultant of a complex combination of absorption and scattering events during the path of light traveling through the skin. A longer path length increases the probability of absorption events and the path length itself depends predominately on scattering properties of the tissue [40]. Besides absorbers or chromophores which are spatially and temporally invariant, other chromophores can change dynamically influenced by physiological processes resulting in slight variations in the color of skin [41]. Melanin, hemoglobin [42] (p. 11) and its derivatives are the most common chromophores in the skin. The perceived color of skin depends on the distribution, concentration and depth of these chromophores. Figure 2 shows a simplified 3-layer model of light-interaction in the various layers of skin. Various layers, like epidermis, dermis and subcutaneous tissue, have different properties regarding scattering and absorption parameters.

Melanin is expected to occur mainly in the epidermis, whereas hemoglobin is predominately found in the dermis layer. The lowest layer subcutaneous tissue optically functions as a reflector of the light that reaches these depths. The spectral absorption of hemoglobin, which is a dominant chromophore in red blood cells, depends on the oxygen levels bound to the hemoglobin molecule [45] (Figure 2). The color of skin is affected by the slight differences in absorption in oxy and deoxygenated hemoglobin and could potentially be measured with optical techniques in non contact.

### 2.2. Characteristics of SFA Cameras

**Figure 3:**
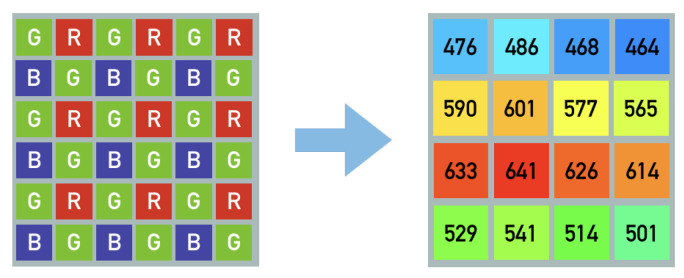
Illustration of common Bayer pattern RGB mosaic and spectral filter array with 16 different peak wavelengths [nm] equally distributed over the whole sensor. The indicated spectral sensitivity peaks show the sensor implementation available for this study.

Digital color cameras utilize color filter arrays in broad red, green and blue ranges of the spectrum, commonly referred to as RGB. A 2 × 2 pixel array mosaic covers a large megapixel sensor containing small red, green and blue filters. Through image processing and demosaicking algorithms a full color image is reconstructed. In case of spectral filter array (SFA) sensors this method is scaled up to larger pixel arrays [15,29] with narrow band filters covering specific spectral bands in the visible (400–650 nm) or the near infrared (650–900 nm range). An example of this is illustrated in Figure 3 showing the peak wavelength and filter mosaic of the commercially available camera used in this study (IMEC [27], XIMEA [46]).

In principle these spectrally sensitive filters can be arranged specifically and various different patterns have been proposed. Demosaicking algorithms allow the reconstruction of a full spectral image cube [15,29,30].

SFA spectral imaging unlike conventional spectral imaging allows to simultaneously acquire all wavelengths at the same time in a snapshot fashion at typical video rates (20–50 fps) [16]. These spectral images enable discrimination of physiological markers in high speed, which have potential for medical diagnostics. However, the selected wavelengths of the SFA filters can be sub-optimal for wavelength of interest in medical diagnostics. Also the filtering might ’leak’ an additional secondary wavelength, complicating the analysis.

Fabry-Pérot filters have two highly reflective coatings on the surfaces of a substrate. The distance between these reflective surfaces is ‘the cavity’. Depending on the thickness of this cavity the transmission through the coatings changes and only a specific wavelength passes to the sensor. SFA filter technology introduces crosstalk of adjacent channels, second order responses and spectral leaking [20,24].

Spectral leaking describes the limitation of reflective surfaces that are only defined in an active spectral region. It can be accounted for by limiting the active range using a band-pass filter mounted on the spectral filter array.

Crosstalk describes the influence of one pixels signal onto a neighboring pixels signal. This especially effects SFA sensors, due to their high spatial variation of filter sensitivities.

Second order harmonics are an inherent hardware limitation of Fabry-Pérot SFAs, due to the spectral filter design. The thickness of the cavity defines the peak wavelength for transmission based on constructive interference of the light trapped in the cavity. But the constructive interference occurs for all light in phase and depends on the wavelength, the angle of light, the thickness of the cavity and the refractive index of the material between the surfaces. Due to the nature of these filters second order peaks also interfere constructively and are therefore transmitted, making the filters sensitive to two wavelength peaks. Some of these additional peaks are filtered out through limiting the active region with a bandpass filter but some of the second order peaks need to be corrected for.

Next to hardware solutions, crosstalk and second order responses can be corrected numerically as a post processing step.

SFA imaging technology requires hardware-aware processing [24,30] both spatially and spectrally to rearrange the pixels of particular spectral sensitivity into an ordered spectral image cube. There are various processing techniques discussed in literature. According to Lapray et al. [20] the filter arrangement should be interconnected with the optimal processing or demosaicking approach in order to obtain the best signal from these kind of sensors. Sadeghipoor et al. [47] propose a spectral demultiplexing of visible and near infrared overlapping spectral information by using spatial and spectral correlation of the channels. This limitation could also be addressed by adding a specific color restoration step into the processing chain as proposed by Park and Kung [48]. These examples of spectral filter array processing discuss different approaches for using SFA data and illustrate the necessity of hardware-aware processing.

### 2.3. Oxygenation Physiology

Oxygenation as a systematic parameter is measured with so called (pulse) oximetry systems [34,49]. Two modes are used clinically, the transmission mode and the reflectance mode. Both modes measure the detected light, which has been altered due to absorption in the tissue. These absorptions depend on physiological properties of the tissue Pulse oximetry measures tissue oxygen saturation as a systematic parameter in one particular spot Non pulsatile oximeters allow the measurement of location based differences. But both are limited to a small measurement area and do not indicate spatial differences of tissue oxygenation.

**Figure 4:**
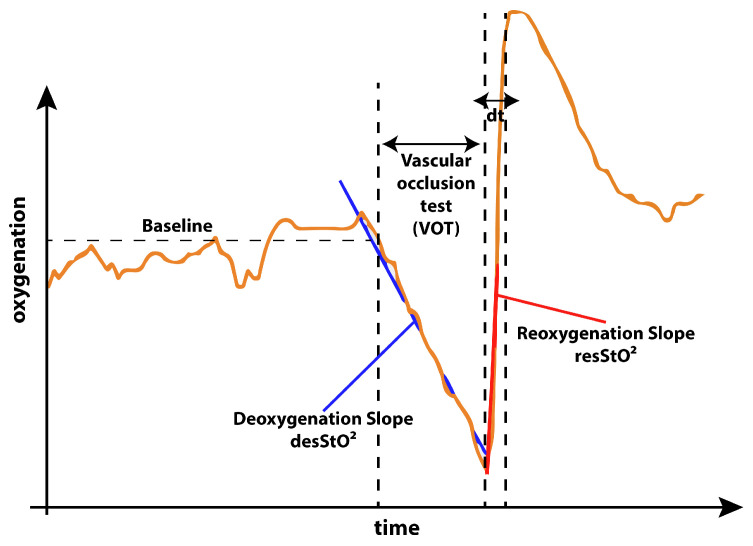
Expected behavior of oxygenation during the occlusion experiment. As described by Futier et al. [50] in the not occluded phase of the experiment a stable baseline with minor fluctuations is expected. As soon as the blood flow is occluded during the vascular occlusion test (VOT), the oxygenation decreases following a deoxygenation slope. As soon as the cuff is released the reoxygenation with an overshoot of oxygenation occurs. The slope providing a way of quantification of the reoxygenation. The time from cuff release to the reoxygenation overshoot peak is quantified as dt. After this the oxygenation curve returns back to baseline.

The well documented [28,31,33] occlusion test behaves as illustrated in Figure 4. A decrease of oxygen concentration during occlusion can be expected followed by a sharp incline with an overshoot of oxygen concentration just after the moment of cuff deflation and followed by a slow return to baseline after the overshoot of oxygen concentration.

For physicians, the characteristics of this oxygenation curve can be correlated to the health status of vasculature of the patient. Influences of anesthesia on these key features of the occlusion test were studied by Bernet et al. [51] and they report that anesthesia have an impact compared to a healthy control group. Lipcsey et al. [52] report indications that NIRS measurements of these parameters can provide information about fluid responsiveness of patients and predict surgical complication and reactions to anesthesia. Abelmalak et al. [53] correlate preoperative values for the discussed parameters with serious post operative complications.

## 3. Methods

### 3.1. Data Acquisition and Processing

The process to obtain a spectral image cube with conventional spectral imaging systems is obvious. Multiple narrowband spectral captures are performed and concatenated to obtain a spectral image cube. Black and white corrections are included in order to account for the scene illumination and spatial differences of the illumination.

SFA cameras, however, need to be processed spatially and spectrally in order to obtain a spectral image cube. For this study a commercially available SFA camera-based on the IMEC [27] snapshot sensor, the XIMEA *XiSpec SM4x4 VIS* [46] operating in the visual range from 470 nm to 630 nm with 16 channels was used.

The acquisition of images was performed with the “*XiCamtool*” tool version 4.7 by XIMEA [46] with settings for frame rate, exposure time (described in Table 2) and the raw (.tiff) file format in sequences. All acquisitions were performed with a constant exposure time (50 ms) chosen to stay within the 66% saturation, where linearity of the sensor is guaranteed by the manufacturer [27]. No further linearization or inverse camera response function was applied and this study does not include a measurement of linearity of the camera. A dark reference $I_{Dn}\left(x,y\right)$ image and a white reference $I_{Wn}\left(x,y\right)$ image were taken with the same optical setup. For the ’white’ reference a grey diffuse reflectance standard (Spectralon SRT-20-100 20%, 10 × 10 inches) was used. The reflectance standard was chosen to be grey rather then white to avoid over saturation for any wavelength compared to the measured skin and in order to stay within the dynamic range of the camera which covers 16 wavelength bands with non-uniform sensitivities.

An illumination corrected image $I_{n}\left(x,y\right)$ can be obtained using this method. Each of the sample images was therefore given by:

(1) $$I_{n}\left(x,y\right)=\frac{I_{Sn}\left(x,y\right)-I_{Dn}\left(x,y\right)}{I_{Wn}\left(x,y\right)-I_{Dn}\left(x,y\right)}$$

This process followed the recommendations by McCann [54] for using diffuse reflectance standards to account for the spectral power distribution and inhomogeneities of the illumination. Previous test measurements of a *Gretag MacBeth* color chart have shown that this flat field correction combined with the spectral correction allows adequate reconstructions of spectral reflectances. The acquisition speed of one fps was chosen in order to exceed the acquisition speed of the INVOS system with one frame every three to four seconds, while at the same time ensuring the reliable recording of the data via USB three connection. Disk space for the consecutive measurements was limited. Reducing the amount of data created per measurement was therefore necessary. This relatively slow acquisition speed (for an SFA camera) is fast enough for this type of oxygenation measurements and three times faster then the reference measurements.

The 1088 × 2018 full frame images consisting of 4 × 4 repeated multispectral grids have to be spatially separated into single spectral channels. This is reducing the spatial resolution to 1/4 resulting in 16 spectrally separated 272 × 512 pixel images in .tiff format that can be converted to .ids video sequence files. These processing steps are incorporated in a custom made program called “Multispec” [55,56] not part of the contribution of this research.

### 3.2. Spectral Correction

The sensitivities of the SFA camera used in this study have significant second order peaks and are overlapping (cross talk) for which a mathematical spectral correction of the data is necessary.

**Figure 5:**
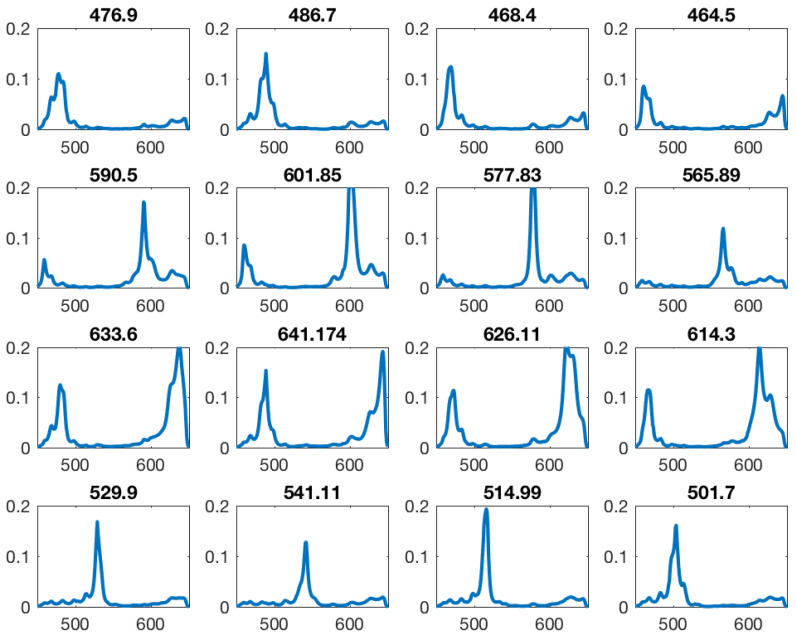
All filter sensitivity multiplied by the bandpass. Many filters show a second order peak inside the sensitive area. Intended peak wavelength shown above of each filter sensitivity curve.

A spectral correction matrix specific for the camera used in this study was supplied by the sensor manufacturer (IMEC [27]). The correction is based on detailed spectral measurements of each specific filter in front of the pixels as shown in Figure 5. These were measured in a monochromator setup by the manufacturer [27] and provided as data together with the camera. They characterize the imperfections of each specific filter.

The spectral correction is intended to minimize the ‘imperfections’ but is not optimized for a specific application. In this research the calibration matrix provided by the manufacturer was used. A calibration matrix can be determined using defined spectral reflectances in form of *Gretag MacBeth* color chart spanning a wide range of different spectral shapes. Essentially this spectral correction is a linear transformation of the cameras imperfect broad sensitivities including second order peaks to idealized virtual narrow band sensitivities. It can be described as a linear transformation and the theory and assumptions were previously described by Connah et al. [57]. Generally the camera response per channel $P_{i}$ is described with reflectance *R* (of any object) under illuminant *E* and channel specific sensitivities $Q_{i}$ and system specific noise *n* with the equation:

(2) $$P_{i}=\int_{\lambda }Q_{i}\left(\lambda \right)E\left(\lambda \right)R\left(\lambda \right)d\lambda +n$$

The previously described illumination correction allows to simplify the equation. After correction for illumination and assuming continuous functions of wavelength sampled at discrete intervals (i.e., 31 measurements) this can be reformulated in matrix form to:

(3) $$p=Q_{e}^{T}r+n$$

The recovered reflectance is described as a spectrum through a given camera response *p*. $Q_{e}$ describes the effective sensor sensitivities. Assuming smooth curves, reflectances *r* can be approximated with the linear combination of a number of basis functions and weighting factors [57],

(4) r≈Bw,

where *B* is the matrix 31×m with *m* different basic functions or number of channels. *w* describes the weights assigned to each of these basic functions in order to approximate *r* best. Then the response *p* is:

(5) $$p\approx Q_{e}^{T}Bw+n,$$

For given reflectances the weights *w* and the basis functions *B* can be determined and the error between the known spectrum and the approximated spectrum minimized. These are calculated as a preprocessing step for known color checker reflectances. This correction is not particularly tuned for skin imaging and can be considered a preprocessing step necessary for this SFA implementation. A pair of known reflectance and its corresponding camera responses are used to minimize the projection from spectral to camera space. The correction matrix similar to the correction matrix provided by the manufacturer [27] is obtained and can be used for correction via a matrix dot product with each measured camera response in order to reconstruct a corrected spectrum. This preprocessing step provides the estimated spectral data and is the final input for the oxygenation estimation.

**Figure 6:**
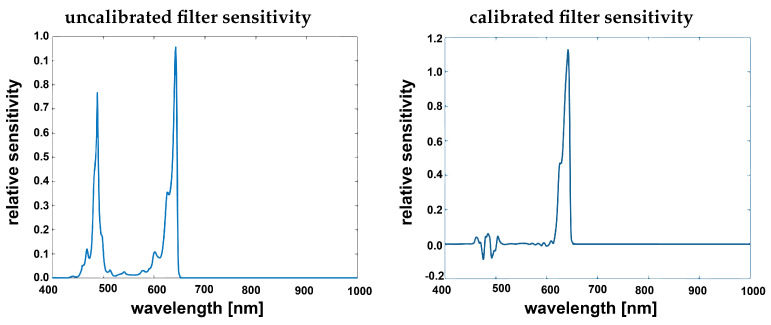
Filter sensitivity given by manufacturer [27] from the calibration file (**left**) filter band-passed showing clear second order harmonics, corrected filter after applying the spectral correction (**right**).

Figure 6 illustrates the effect of this correction, it was applied to one of the filter responses. This illustrates how the spectral correction matrix corrects for the second order peaks and spectral crosstalk.

**Figure 7:**
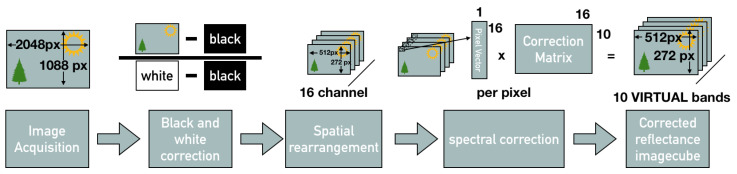
Overview of the processing chain: Image acquisition of the full frame image with the spatial spectral mosaic, full frame black and ’white’ correction, spatial rearrangement of the channels, spectral correction resulting in the reflectance image cube.

The spectral correction transforms from the camera responses to a reflectance image cube and reduces the number of channels from 16 to 10 effective virtual channels, which is similar to the basis functions described by Connah et al. [57]. The correction matrix provided by the manufacturer [27] indicates that the crosstalk in the 600+ nm channels is too severe to use them or reconstruct them numerically. A custom reconstruction matrix could be calculated and estimate results for these bands as described. The resulting spectral image cube with 10 specific bands can then be used for further processing and analysis. This processing step is integrated into the proposed processing chain and an overview is provided in Figure 7. All of these bands can be used depending on the medical application and the specific bands of interest.

### 3.3. Oxygenation Estimation from a Multispectral Image Cube

As described above the SFA camera enables spectral images in 10 narrow wavelength bands of the scene after correction. To estimate oxygenation from these spectral images, the previously proposed method called the Δt method [58,59,60] was applied. This method has been chosen due to low computational complexity and in order to enable real time estimation of oxygenation of the tissue in the future. And furthermore since it is the most commonly used technique for fNIR spectroscopy, used for oximetry systems. This allows to maintain a high degree of comparability between the INVOS^TM^ oximetry reference system and the SFA camera test system.

In its current implementation this method utilises only three key wavelength chosen based on their descriptive nature of the absorption spectrum of oxy and deoxygenated hemoglobin. Other wavelength imaged with the SFA setup are not utilized for the application of oxygen estimation in the current implementation.

The Δt method is based on the modified Beer-Lambert law and considers absorption and scattering as the main reasons for the attenuation of light in tissue. The method assumes that the absorption can be separated in two parts: (1) a time invariable constant absorption due to chromophores present in the skin and (2) a time variant absorption due to changing oxy and deoxygenation in the skin. These depend both on the oxygen concentration and the total blood volume present during measurement. Optical density or absorption can be defined as,

(6) $$OD=-log_{10}\left(\frac{I}{I_{0}}\right)=\sum_{n}\varepsilon_{n}*c_{n}*d;$$

where OD stands for optical density, $I_{0}$ is the emitted light intensity and *I* is the intensity of the received light, ε describes the molar extinction coefficient for *n* different chromophores, *c* is the concentration of the chromophore *n* and *d* the path length taken by the light. The modified Beer-Lambert law can describe the longer path-length of light through the medium, due to scattering [61] as,

(7) $$\bar{A(\lambda )}=\bar{\varepsilon (\lambda )}*\bar{c(t)}*\bar{DPF(\lambda )}*d+\bar{G(\lambda )}+\bar{H(t)};$$

where $\bar{A(\lambda )}$ is the absorbance, $\bar{\varepsilon (\lambda )}$ describes the molar extinction coefficient [mM^−1^ cm^−1^], $\bar{c(t)}$ is the concentration of a specific chromophore [mM], $\bar{DPF(\lambda )}$ differential path length factor corrects the geometrical source-detector distance to the mean optical path in the tissue, *d* the source detector distance [cm] and $\bar{G(\lambda )}$ and $\bar{H(t)}$ are both oxygen independent loss factors accounting for scattering, absorption and geometry losses where $\bar{H(t)}$ is time dependent and $\bar{G(\lambda )}$ is wavelength dependent.

This can be rewritten in matrix form and with three specific wavelengths to:

(8) $$\left[\begin{array}{c} A(\lambda_{1},t)\\ A(\lambda_{2},t)\\ A(\lambda_{3},t) \end{array}\right]=\left[\begin{array}{cc} \varepsilon_{O_{2}Hb}\left(\lambda_{1}\right)DPF(\lambda_{1}) & \varepsilon_{HHb}\left(\lambda_{1}\right)DPF(\lambda_{1})\\ \varepsilon_{O_{2}Hb}\left(\lambda_{2}\right)DPF(\lambda_{2}) & \varepsilon_{HHb}\left(\lambda_{2}\right)DPF(\lambda_{2})\\ \varepsilon_{O_{2}Hb}\left(\lambda_{3}\right)DPF(\lambda_{3}) & \varepsilon_{HHb}\left(\lambda_{3}\right)DPF(\lambda_{3}) \end{array}\right]*\left[\begin{array}{c} c_{O_{2}Hb}\left(t\right)\\ c_{HHb}\left(t\right) \end{array}\right]d+\left[\begin{array}{c} G(\lambda_{1})\\ G(\lambda_{2})\\ G(\lambda_{3}) \end{array}\right]+\left[\begin{array}{c} H(t)\\ H(t)\\ H(t) \end{array}\right]$$

The Factor *G* is accounting for geometry losses and due to the fact that the measurement geometry is kept constant it can be assumed that *G* stays constant throughout the measurement. The *H* term can be considered zero or constant in time, since no significant changes in the optical properties of the skin are to be expected in the timespan of this experiment. $\varepsilon_{O_{2}Hb}$ and $\varepsilon_{HHb}$ describe the unique absorbances of the oxygenated hemoglobin and deoxygenated hemoglobin.

The difference in oxygen concentration at a time point (Δt) relative to a stable starting point can be calculated by simplifying the equation to:

(9) $$\Delta_{t}\bar{c}=\frac{\bar{\varepsilon }{\bar{DPF}}^{-1}\Delta_{t}\bar{A}}{d}$$

**Figure 8:**
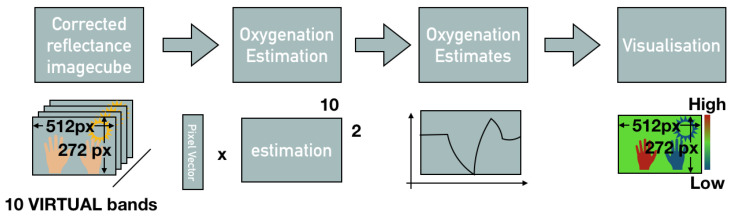
Oxygenation estimation from spectral reflectance cube.

The equation contains $\bar{DPF(\lambda )}$ values from literature for the specific interrogated tissue. This formula allows to relate changes in light intensity of particular wavelength to changes in oxy, deoxygenated hemoglobin concentrations. The average of the first few frames of the measurement are used to estimate the time invariant contributions to the measured skin reflectance spectrum. Changes of the skin spectrum especially in the areas of highest absorption differences between oxy, deoxygenated hemoglobin are calculated. This oxygen concentration estimation can be applied using three wavelength (515 nm, 565 nm and 601 nm) from the reflectance image cubes previously obtained with the SFA camera and the process is illustrated in Figure 8.

**Figure 9:**
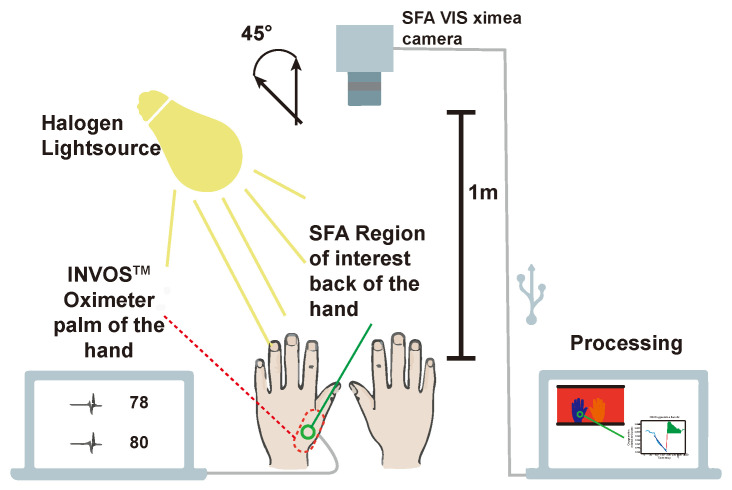
Experimental setup. XIMEA [46] camera 1m above volunteers hands. 45° (following the CIE recommendation [62] and ([45] p. 144)) between halogen lightsource and SFA camera. Parallel measurement at the palm of the hand with the INVOS. SFA measures at the back of the hand with green circle indicating region of interest for averaging. External processing on a computer (adapted from Reference [39]).

These three wavelengths are specifically chosen, based on the spectral absorption peaks and differences in absorption of oxygenated and deoxygenated hemoglobin. They contain descriptors of the oxygenation with a small difference at 515 nm and large differences at 565 nm and 601 nm in absorption between two oxygenation states of hemoglobin. The small difference point provides a reference (isosbestic point in the visual range), while the points of large difference provide good estimators [28]. Even though the estimation is performed for the entire image only an average of the estimated oxygenation in the marked region of interest as shown in Figure 9 (green circle) was collected. The resulting oxygenation and estimated oxygenation curves from the INVOS and the multispectral system were marked by collaborating physicians for the moment of pressure release in the curves of both measurement devices. Since the SFA based oxygenation estimates are of relative unit of small scale a feature scaling normalization had to be applied to both curves:

(10) $$X^{\prime }=\frac{X-X_{min}}{X_{max}-X_{min}};$$

After normalization both curves are in the value range of [0, 1] allowing comparison between the curves. This feature scaling affects the amplitude of the oxygenation signals and favors the comparability between the two curves. Nevertheless this processing step is necessary to compare the shapes and oxygenation behavior measure with both devices. As another processing step the curves are aligned along the time axis using the markers for cuff release as the minimum point of both curves.

The oximeter samples the oxygenation significantly slower, with only one measurement every three to four seconds compared to the SFA setup with 1 frame per second. Therefore, a moving average of three values for the SFA oxygenation estimates was taken in order to resemble the sample rate of the INVOS oximeter better. These steps and the following data analysis and visualization was performed using custom written *python* code.

### 3.4. Proof of Concept Oxygenation Study

For proof of concept an in vivo study was performed on human volunteers. Both the proposed SFA setup and the clinical reference (gold standard) were used. The well documented upper arm occlusion [28,31,33] test was chosen for comparison. The SFA camera XIMEA^TM^ [46] *XiSpec SM4x4 VIS* was used.

An inflatable cuff was used on one of the arms of a volunteer. The arm is clamped for approximately three minutes decreasing the blood-flow significantly resulting in a decrease of oxygenation by around 60%. The standard oxygenation measurement is performed with a sensor taped on the skin emitting two wavelengths and sensing the ratio of the reflected light. An overview of the experimental setup is provided in Figure 9.

The camera was placed in 1 m distance from the hands of the volunteers. Skin can be considered a rough surface and contains an oily layer. The skin of the hands was illuminated under an angle of 45° in order to minimize the specular reflection from the tissue surface. The camera sensor plane was parallel (0°) to the upper side of the hands. This way a 0/45° experimental setup following the CIE recommendation [62] for color measurements and the practice for white light spectroscopy in the medical field [45] (p. 144) were followed.

The INVOS “Cerebral/Somatic Oximetry Adult Sensor” was positioned on the palm of the hand out of view for the camera. By the region of interest (ROI) for the SFA oxygenation measurement was chosen close to the position of the INVOS sensor.

All 58 volunteers had no reported previous condition or vascular diseases and were in the age group of 18–65 years old. The experiment was approved by the Medical Ethics Committee of the VU University Medical Center (METc-16.315). Written informed consent was obtained from all 58 volunteers prior to participation.

The baseline blood pressure and temperature of the volunteers were measured prior to the experiment. An inflatable cuff was placed around the left upper arm and inflated to 30 mmHg above the previously measured systolic blood pressure. The cuff was kept inflated until the reading of the INVOS^TM^ NIRS system dropped to 40% of baseline oxygen saturation following the protocol described by Meyer et al. [63]. As soon as this value was reached the cuff was immediately deflated and the blood perfusion in the arm and hand were restored.

Both the moment when the occlusion started and the moment when the occlusion was stopped were marked manually for both measuring systems in order to later align the time axis adequately. The aligning and calculation of the parameters was based on numerically determined extreme points of lowest detected oxygen saturation and highest detected saturation and the baseline labelled by physicians involved in the study. The highest detected oxygen saturation was only determined in a window where the curve was considered to return to the baseline, this window was also determined by the physicians. For the SFA measurement a reflective marker was added to the scene and removed at the moment the pressure cuff was released. During the occlusion protocol SFA images were acquired continuously.

## 4. Results and Discussion

### 4.1. Spectral Correction and Spatial Down Sampling

An overview of the complete processing chain for SFA imaging is provided in Figure 7 including processing steps for calibration, corrections and analysis. This processing allows to reconstruct the full spectral cube from SFA images. They provide a selection of 10 narrow band wavelength regions that can be used for clinical monitoring and diagnostics.

**Figure 10:**
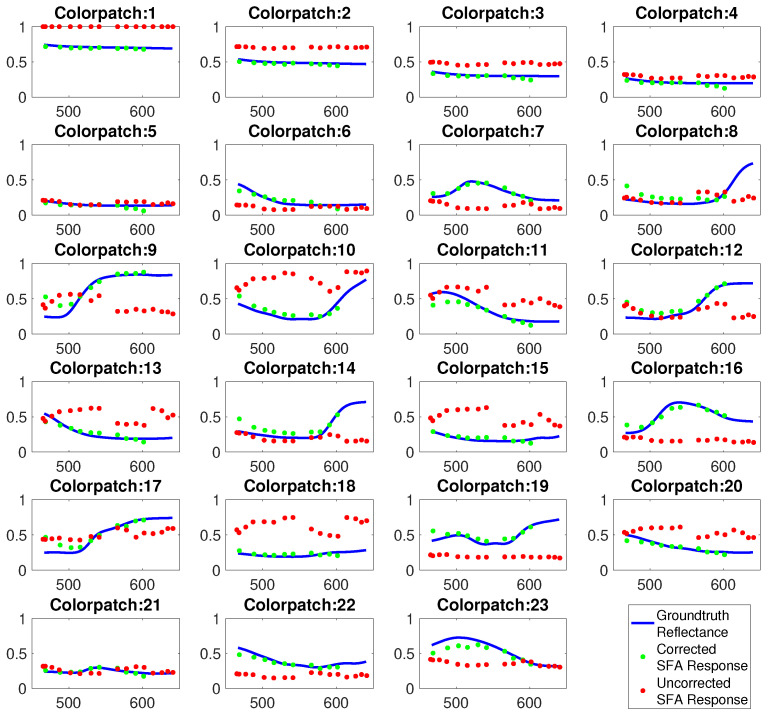
Spectral reflectance of *Gretag MacBeth* color patches measured by full spectral camera (blue), uncorrected spectral estimates of SFA setup (red), spectral corrected estimates (green).

For validation the *Gretag MacBeth* color chart was imaged with a reference spectral camera (SPECIM IQ) and the SFA imaging setup. Figure 10 shows the resulting reflectances of the color chart before and after spectral correction. This illustrates the improved fit with the reference after correction and emphasizes the usefulness of the spectral reconstruction. After correction ten spectral bands are available, a selection can be used containing the relevant pathological or physiologic information.

The spectral correction could be optimized by including prior knowledge about the expected spectra imaged by the SFA system. A dynamic spectral correction could be implemented to provide a better spectral reconstruction. The careful selection of adequate training data more specialized to the skin application might provide better spectral reconstructions of these skin spectra. This would allow the matrix to emphasize subtle changes of skin reflectance. An optimal set of spectral bands for a given application can be determined and implemented in the correction matrix. For oxygenation measurements, a custom built spectral camera only sensitive in three narrow wavelengths bands could improve the applicability. Optimizing the number and distribution of channels numerically could further improve the results.

The straightforward spatial demosaicking reduces the spatial information significantly. In this research the spatial resolution was not further utilized and was not a priority. For applications that need better spatial resolution more advanced techniques to reconstruct missing spatial information exist. These techniques utilize the spatial redundancy as shown in other studies [15,20,29,30].

### 4.2. Proof of Concept Oxygenation Study

**Figure 11:**
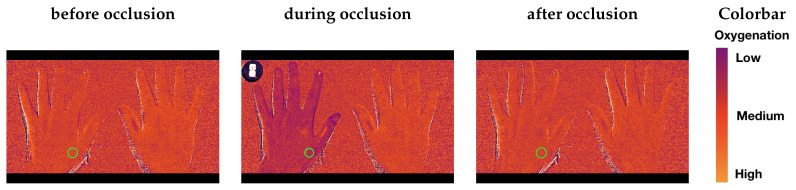
Colormaps of the three different stages of the occlusion before the occlusion (**left**), during the occlusion shortly before the deflation of the cuff (**middle**), reperfusion after the cuff is released (**right**). Green circle indicating the averaged region of interest for oxygenation curves.

Figure 11 illustrates the color maps at three different stages of the occlusion protocol that are generated using the SFA data collected. Spatial differences along the surface of the hand are visible, blue indicating low oxygenation and red indicating higher oxygenation. The right hand is shown as a reference for validation of the measurement and is not occluded. The clinical reference INVOS 5100C-PA provides a local measurements on the palm of the hand, opposite to the region of interest for the SFA system (indicated with a green circle).

**Figure 12:**
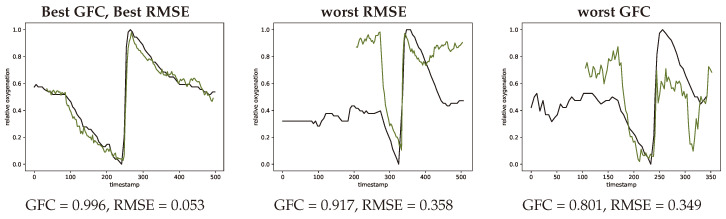
Extreme curves with best GFC and best RMSE (**left**, #V56-1) worst RMSE (**middle**, #V07-1) and worst GFC (**right**, #V39-1) metrics. INVOS measurement in black and SFA estimation in green.

In order to compare the shapes of the oxygenation curves root mean square error (RMSE) and goodness of fit coefficients (GFC) were calculated. These compare the general shape of the two curves and provide a metric for their agreement or similarity according to their shapes. In Figure 12 representative normalized oxygenation curves with lowest root mean square error and best goodness of fit coefficients and the respectively lowest performing examples according to these two metrics.

**Figure 13:**
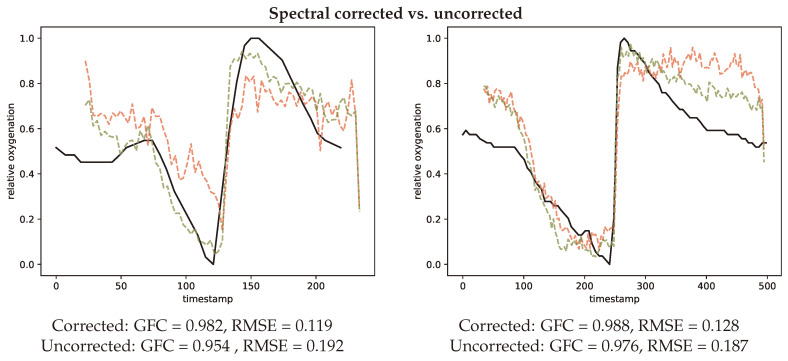
Two representative curves (**left**, #V03-1) (**right**, #V56-1) comparing spectral correction (green) and spectral uncorrected (red) oxygenation estimations in comparison to the INVOS measurement (black).

Figure 13 contains three representative plots illustrating the impact of different aspects in the proposed processing pipeline. The top row illustrates the impact of the spectral correction step for two example volunteers. In both cases it can be observed that the agreement between ground truth measurement (black) and estimated oxygenation improves with the spectral correction. For volunteer one (left) RMSE improves by 0.73 and GFC by 0.28 for the second volunteer (right) the changes are GFC = 0.14 and RMSE = 0.59.

**Figure 14:**
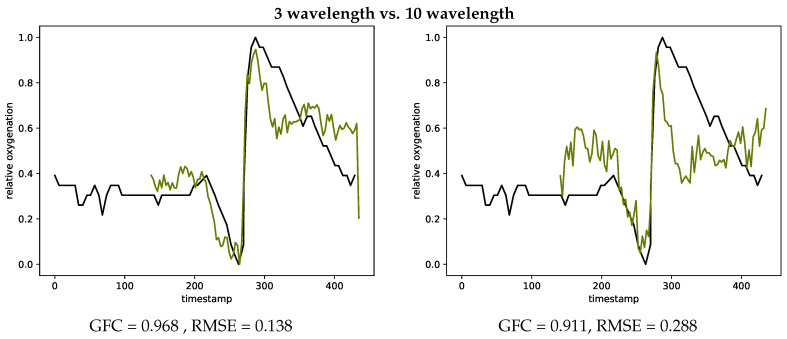
Oxygenation curves calculated with the three chosen wavelength (**left**, #V27-2) and using all wavelength (**right**, #V27-2) in comparison with the INVOS measurements.

Figure 14 also provides examples showing the difference of using three chosen wavelength (left) compared to all wavelength (right) for estimating the oxygenation curves. The metrics GFC and RMSE both result in a smaller agreement with the gold standard INVOS measurement devices, when using all wavelength.

Table 3 presents the statistical data for all measurements on the volunteers.

Considering the GFC and RMSE metrics there is an acceptable agreement with an average GFC of 0.965 and 0.185 for RMSE, the SFA imaging technique shows to be promising for spatial oxygenation measurements.

**Figure 15:**
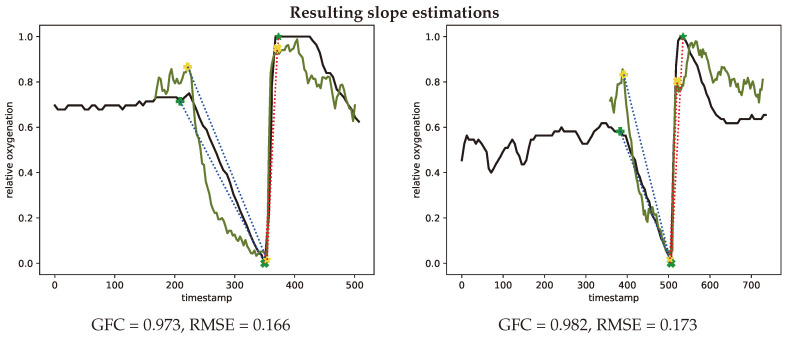
Representative oxygenation curves from the INVOS system (black) and estimates from the spectral filter array system (green). Linear desaturation (blue dots) between baseline (+) and low point of oxygenation (x (SFA) and * (INVOS)). Linear resaturation (red dots) between low point (x (SFA) and * (INVOS)) and high point (x (SFA) and * (INVOS)) of oxygenation. Representative curves with median GFC (**left**, #V46-2) and median RMSE (**right**, #V01-1) metrics.

The de- and re-saturation slopes are parameters of interest for a preoperative or anesthesiological context. These slopes were determined from the saturation curves using linear fitting, the de-saturation slope in blue and re-saturation slope in red, this is illustrated in Figure 15 for both the INVOS system and the SFA setup.

**Figure 16:**
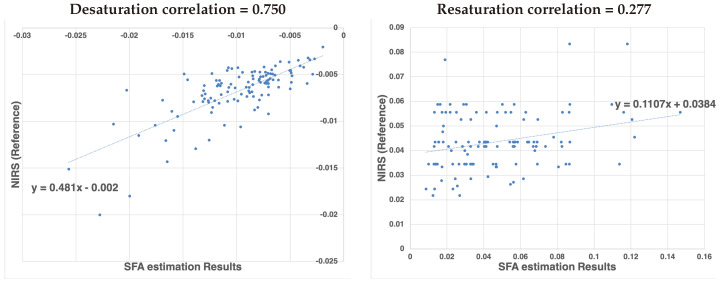
Correlation of de-saturation (**left**) and re-saturation (**right**) slope numbers between the proposed SFA setup and the clinical standard NIRS system.

In Figure 16 the calculated de- and re-saturation slopes from the reference system compared to the SFA setup are presented for all measurement showing a sample (Pearson) correlation coefficient of 0.750 for de-saturation and 0.276 for re-saturation.

The significantly lower correlation for the re-saturation slope might be ascribed to the method of analysis. The begin and end point of the steep re-saturation slope were determined numerically as the minimum and maximum in a manually defined area. A small shift in the position of these points results in a larger variation between measurement devices.

The difference in measurement technique between the INVOS system and the proposed SFA system need to be considered. The INVOS provides absolute oxygenation measurements in contrast to the oxygenation related relative estimates from the SFA setup. These estimates are closely related to oxygenation as shown in Figure 15 but may be influenced by other physiological effects in addition. One of these effects could be blood volume, which might not be adequately accounted for. As mentioned in Section 3.3 the SFA oxygenation estimation utilizes the first frames to account for time invariant optical properties in order to estimate oxygenation changes. The total blood volume is a time variant property and could be estimated incorrectly in those first key frames.

The INVOS and SFA setup use different wavelength regimes, which represents different sampling volumes due to penetration depth depending on wavelength. Therefore, the SFA system measures more superficially compared to the INVOS system. The occlusion certainly blocks the blood distribution to both layers but the response in oxygen consumption might differ. For the upper arm occlusion following the proposed protocol the expected difference mainly affects the amplitude of oxygenation changes. Since the oxygen consumption is different in the two sampled volumes.

The normalization used for this work could positively affect the correlations between the visual range camera and the near infrared INVOS system. The feature scaling reduces the differences in the measured amplitude. The clinical gold standard has been used as a validation that the SFA system senses oxygenation concentration changes. The sample (Pearson) correlation coefficient between the reference measurement and the SFA system can be considered as an indication that the SFA setup measures these changes. Further investigation into the difference of sampling depth has to be carried out.

The oxygenation estimation with the SFA setup is based on three key wavelength. Results could be improved by using the full spectral curve for oxygen estimation and finding a fit to previously simulated spectra. Using all wavelength showed no improvement for the considered method in this research as shown in Figure 14. An optimization of the band selection could improve the oxygenation estimation results. Bjorgen et al. [64] proposed a method for real time processing following this principle. Vyas et al. [65] combine Kubelka Munk forward modeling with machine learning approaches to estimate the chromophore concentrations from spectral signatures.

With further processing absolute oxygenation values could be determined. This study providing a ‘proof of concept’ is not at the stage to derive absolute oxygenation values. It provides a starting point by combining spectral correction and medically relevant processing of the measured spectra for further investigation.

### 4.3. Practical Use of SFA in a Clinical Environment

SFA cameras hold the potential to be a practical devices for medical applications given their acquisition speed, spatial and spectral acquisition properties, device size, potential ease of use and application versatility as a non-contact measurement method.

The aim of this study was to propose solutions for the technical limitations of this new kind of spectral imager while maintaining the full spectral, temporal and spatial capabilities. The current clinical gold standard for measuring oxygenation is limited to slow point measurements in direct contact to the patient impeding the applicability for burn-wound assessment, wound healing, neonatal units, local control measurements or even large scale operating room oxygenation monitoring. SFA cameras have the potential to improve upon the clinical gold standard for oxygenation measurements by non contact measurements with a spatial resolution and fast acquisition speed. In order to measure the oxygenation of burn-wounds a near infrared model of the camera might be necessary. Previous work [39] has shown that the proposed method for oxygen estimation can be applied to the near infrared version of the camera.

A step by step processing pipeline including black and white correction, spatial rearrangement and spectral correction and oxygenation estimation has been implemented. It was tested with an in vivo volunteer experiment using an existing commercially available SFA camera. The process can be generalized and used for other SFA cameras if the spectral sensitivities are known. This pipeline has been implemented in consideration of real time processing and requires little optimizations for a real time implementation. The matrix operations for the spectral calibration could be performed on the fly when implemented on a Field-programmable gate array (FPGA) added between the camera and the computer for processing. White and dark correction could be added as a calibration procedure before starting to use the camera. The oxygenation estimation in its simplified version can be implemented for real time application if incorporated into the acquisition program.

In the future less crosstalk between the channels would be desirable. The wavelength range is of importance depending on the application and a combination of both near infrared and visual wavelengths regimes would extent the use cases. Lower prices and wider adoption of SFA technology can be expected and should assist to mature this versatile and new technology for the medical field.

This study also shows the different aspects in which the processing of SFA cameras can be improved including optimal band selection, spectral correction and spatial processing and highlights the impact of specific steps on the final oxygenation estimation results.

### 4.4. Comparison to Related Work

Some studies [66] including Spigulis and Oshina [67] propose the estimation of absolute oxygenation values from an RGB imaging setup. An area of the skin is illuminated with different monochromatic laser light sources. Using an RGB sensor an image of the scene is acquired and a multi band image cube generated from the differently illuminated areas. This is a low-cost way of obtaining medically relevant narrow band channels but it requires a custom made illumination setup in conjunction with optimally chosen RGB sensor sensitivities.

Saager et al. [68] provide a theoretical framework for evaluation of the capability of different multispectral imaging techniques including spectral filter arrays to quantify chromophores in the context of burn wound healing. They consider the same *XiSpec* SFA camera implementation on a theoretical level with an existing skin and phantom database. Simulated spectra were generated using the spectral sensitivities of the *XiSpec* SFA camera. Saager and his colleagues conclude that the camera provides reasonable accuracy for most common chromophores. Even-though, to the best of our knowledge the discussed spectral correction see Section 3.2 to account for double peaks was not applied to their theoretical data analysis. This stresses the indication of potential for SFA cameras as quantification tool for oxygenation related health metrics, since it could improve the resulting oxygen estimations.

Ewerløf et al. [28] propose the use of the same SFA camera for oxygenation estimation using an inverse Monte Carlo modeling. A database of Monte Carlo simulated spectra with known optical properties is prepared. The database of spectra is then multiplied by the sensor sensitivities and the illumination. This new database can then be used to estimate optical properties from measurements with the camera, by minimizing the difference between the pre simulated data and the measured camera responses. All channels measured by the camera are used for the estimation. It is not applying the spectral reconstruction and some of the additional channels might be introducing noise, due to their overlapping nature. While this method does not require the proposed spectral calibration, it requires prior Monte Carlo simulations adequately representing the patient population.

## 5. Conclusions

The feasibility of a commercially available SFA camera for clinical applications is tested. This study proposes a basic processing pipeline to solve shortcomings and challenges of this new spectral imaging technology. The pipeline maintains spectral, spatial and temporal capabilities of a commercially available SFA camera and is directly transferable to other SFA cameras. Technical challenges and indications by numerically correcting for double lobes in the spectral sensitivities have to be managed with care and a hardware based solution is advisable. SFA cameras and their benefits in a medical context have been studied by a proof of concept in vivo voluntary oxygenation experiment including 58 volunteers and 116 measurements. Results obtained have been validated with the clinical standard for oxygen measurements and promising agreement for the shapes of oxygenation curves were shown. The medically relevant parameters for desaturation and resaturation slopes show moderate correlations, which can be improved upon. This moderate correlation can be ascribed to slight differences in measurement frequency, difference in sampling depth and the strong impact of small differences for the calculated slopes. Aspects of the proposed processing need to be further improved including the spectral correction, real time processing, oxygenation estimation and real time visualizations.

## Figures and Tables

**Table 1:** Overview of spectral imaging techniques including RGB [33], filter wheel cameras (FW), liquid crystal tunable filter (LCTF) cameras, spectroscopy [34] (Spec), spatial frequency domain imaging (SFDI) [31,35,36,37], multispectral illumination (MI) [1,38], SFA spectral filter array [39].

Property	RGB	FW	LCTF	Spec	SFDI	MI	SFA
spatial acquisition	2D	2D	2D	point	2D	2D	2D
spectral bands	3	8–10	100	x > 100	x > 100	∼20	8–16
spectral acquisition	snapshot	sequential	sequential	snapshot	sequential	sequential	snapshot
frame rate	∼150 fps	∼60 fps	∼1 f\0.05 s	∼1 fps	∼60 fps	∼1 f\0.05 s	∼30 fps
cost	low	medium	medium	low	medium	medium	medium
Processing complexity	low	low	medium	low	medium	medium	medium
effort of use	low	medium	medium	low	medium	medium	low

**Table 2:** Camera parameters chosen for the acquisition.

Camera Property	Value
Acquisition speed	1 fps
Exposure time	50 ms
Aperture	2.8 f
Lense	Edmund Optics 35 mm C Series VIS-NIR

**Table 3:** Minimum, maximum, average, standard deviation and 95 percentile values for both root mean square error and goodness of fit coefficient. Small root mean square error corresponds to desired value and close to one corresponds to a desired goodness of fit coefficient value.

	Min	Max	Avg	Med	Std	95%
RMSE	0.053	0.358	0.185	0.173	0.064	0.099
GFC	0.801	0.996	0.965	0.973	0.030	0.912

## Abbreviations

The following abbreviations are used in this manuscript:

SFA	Spectral filter array
NIRS	near infrared reflectance spectroscopy
VOT	vascular occlusion test
VIS	visual range
ROI	region of interest
GFC	goodness of fit coefficient
RMSE	root mean square error
